# Outdoor Warehouse Management: UAS-Driven Precision Tracking of Stacked Steel Bars

**DOI:** 10.1007/s42979-025-04206-8

**Published:** 2025-07-28

**Authors:** Assia Belbachir, Antonio M. Ortiz, Erik T. Hauge, Ahmed Nabil Belbachir, Giusy Bonanno, Emanuele Ciccia, Giorgio Felline

**Affiliations:** 1 NORCE Research AS, Grimstad, Norway; 2 ABS Acciaierie Bertoli Safau, Pozzuolo del Friuli, Italy

**Keywords:** Warehouse, Stock-monitoring, Self-products positioning, Drone

## Abstract

Accurately identifying the positions of products in outdoor environments-such as warehouses or industrial yards-presents unique challenges due to variable lighting, weather conditions, and the lack of fixed infrastructure. This work presents a vision-based drone system for product localization using QR code detection and relative positioning. The proposed system enables a UAV to autonomously scan an area, extract QR codes from captured video frames, and compute the spatial relationships between products using a trust-ability graph. Unlike traditional GPS- or RFID-based methods, our approach does not rely on external infrastructure, making it scalable and adaptable for outdoor and semi-structured environments. We demonstrate that the proposed algorithm achieves over 94% positioning accuracy in indoor settings and 80% in outdoor environments, even under occlusion and varying illumination. The key contributions of this work include: (1) a novel infrastructure-free method for product positioning based on relative spatial relationships, (2) the integration of trust-ability scoring to improve the reliability of detected positions, and (3) an extensive evaluation in real-world indoor and outdoor industrial scenarios. These results validate the potential of UAV-assisted inventory systems to enhance automation in logistics and warehouse management.

## Introduction

The ability to automatically monitor product positions within industrial environments is a critical component for efficient inventory management and operational workflow. This article addresses the challenges and solutions in this domain, particularly focusing on the evolution of product positioning techniques and the introduction of a novel relative positioning system.

**Fig. 1:**
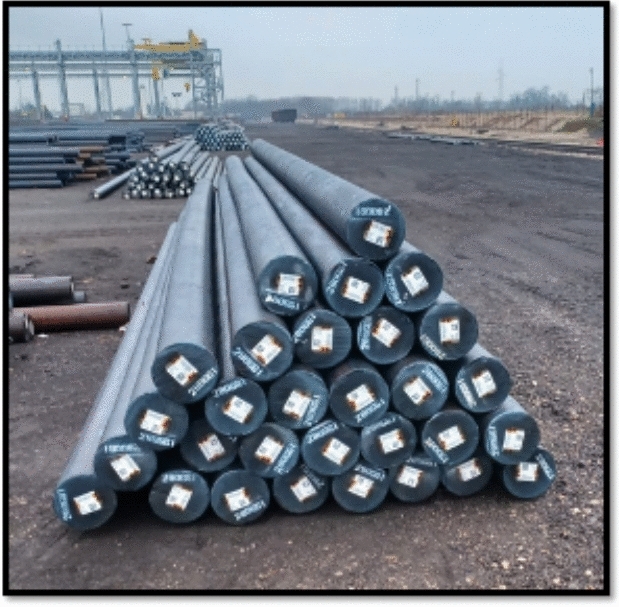
Illustration of the products’ positions with QR code tags

In the context of our work, we consider steel bars (products) that are stacked on top of each other in several ground locations (see Fig. 1). Each group of steel bars has a unique identifier. The bars are stored outdoors, and forklifts equipped with magnetic sensors are used to pick up and relocate them. This picking process often results in interference and small positional shifts. Due to these continuous changes, a human operator must manually re-scan each product, which is time-consuming and labor-intensive.

Historically, industrial settings have relied on diverse technologies to track and position products. Methods such as Radio-Frequency Identification (RFID) have been widely adopted due to their non-line-of-sight data transmission capabilities [8]. However, these systems often face limitations in environments with high metal content or signal interference, leading to inaccuracies in contexts like ours [4].

Similarly, bar-code and QR code systems offer cost-effective solutions but require direct line-of-sight and manual effort. As noted in [11], large-scale scanning can be burdensome for human operators. Advanced systems such as Real-Time Location Systems (RTLS) and GPS-based tracking [10] are designed for broader coverage, yet they typically fall short when precise localization is required.

To overcome these challenges, researchers have explored drone-based systems. For example, [3] proposed a multi-robot system using micro-drones for automated indoor inventory, significantly reducing scanning time. A broader review in [9] examines the growing use of UAVs in warehouse management, highlighting their value in inspection, surveillance, and logistics.

However, ensuring uninterrupted tracking of a drone’s position and accurately correlating drone perception to real-world product locations remains a key challenge. Most existing solutions are heavily dependent on external infrastructure or absolute positioning systems, which are unsuitable for dynamic and outdoor industrial settings.

In this work, we introduce a novel vision-based framework that enables relative product localization using drone-captured video, automatic QR code detection, and product positioning recognition. Rather than relying on fixed reference points or GPS, our approach determines the position of each product relative to its neighbors through a directed graph structure enriched with a trust-ability score. This graph provides both a visual representation of relative positions and a quantitative measure of confidence for each detected relation.

The proposed system is designed to operate autonomously, using UAVs to scan product areas and compute spatial relationships in real time. This eliminates the need for constant human supervision and offers a scalable, infrastructure-free solution for outdoor industrial sites.

The main contributions of this work are as follows:

- We propose a novel drone-based relative positioning system that determines product locations using only onboard vision and QR codes, without relying on GPS or external anchors.
- We introduce a graph-based trust-ability model to quantify the reliability of spatial relations between detected products.
- We implement a complete autonomous pipeline including frame preprocessing, QR code detection, relative graph construction, and visualization of spatial relationships.
- We conduct extensive experiments in both indoor and outdoor industrial-like settings. The results show that our system achieves over 94% positioning accuracy indoors and over 80% outdoors, with high QR code detection rates even under environmental challenges.

This approach lays the foundation for more intelligent warehouse automation systems by reducing dependence on fixed infrastructure and manual labor while increasing robustness in dynamic, real-world environments.

The remainder of this work is organized as follows: Sect. "State of the art" reviews related work on system positioning. Section "Framework for Self-Product Positioning and Trustworthiness" describes the proposed framework and the main concepts involved. Implementation and empirical results are presented in Sect. "Experimental Results", and Sect. "Conclusion" concludes the article and outlines future directions.

## State of the art

Diverse approaches have been proposed to address the challenges of precise positioning and tracking in industrial and warehouse environments. These range from traditional methods such as RFID and barcode systems to advanced drone-based localization technologies. In this section, we discuss significant related work, highlighting their contributions, limitations, and how the work presented in this article advances the current state of the art.

Foundational work on RFID systems in industrial settings is presented in [4, 8], highlighting their efficiency in product tracking due to non-line-of-sight capabilities. However, these systems suffer from performance degradation in metallic environments, a critical drawback in steel bar storage yards.

de Seta [11] explores QR code applications and infrastructural challenges. Although cost-effective and simple to deploy, QR-based systems are hindered by the need for direct line-of-sight and manual scanning.

The work in [10] evaluates Real-Time Locating Systems (RTLS) for production management. While RTLS offers reasonable accuracy, it requires extensive infrastructure and lacks scalability in open or semi-structured environments.

Davide [3] proposed the use of micro-drones for inventory scanning, demonstrating reduced task duration and improved agility. However, their system primarily focused on indoor environments and did not address the complexities of outdoor steel bar storage or relative product positioning.

A systematic literature review of UAV integration in warehouse management was presented in [9], exploring the potential for UAVs in inventory, inspection, and surveillance tasks, yet acknowledging the unresolved issues in positioning accuracy, especially in outdoor and cluttered environments.

The PILOT system by [5] implements drone localization via Time-of-Arrival (ToA) analysis and Frequency-Hopping Spread Spectrum (FHSS). While innovative, it is confined to indoor usage and lacks mechanisms for inter-product spatial relationships.

GNSS-compass fusion approaches such as those presented in [2] improve outdoor navigation but fall short in environments with dense product stacking, where precise intra-warehouse localization is needed.

The use of stereo vision techniques for indoor drone control, as shown in [7], emphasizes 3D reconstruction through drone-mounted cameras. This method boasts high positional accuracy for indoor navigation but offers limited applicability in large outdoor industrial sites.

**Table 1:** Comparison of existing positioning technologies

Method	Accuracy	Infrastructure	Scalability
RFID [4]	10–50 cm	High (readers + tags)	High
QR (manual) [11]	High (manual)	Low	Low
RTLS [10]	20–50 cm	High (nodes)	Medium
UWB [12]	1–5 cm	High (anchors)	Medium
Stereo Vision [7]	High (no quantified data)	Medium (drones)	Low
Drone-based (FHSS) [5]	Not specified	Medium	Low (indoors)

Other approaches, such as Sensor Fusion, integrate ultrasound, LIDAR Time of Flight (ToF), visual odometry, and Ultra-Wide Band (UWB) positioning [12], promising approximately 5 cm accuracy during flight. Furthermore, UWB systems using impulse-radio (IR-UWB) two-way ranging (TWR) can reach 1.2 cm accuracy in semi-closed environments, though they demand dense anchor deployment.

Fernandez [6] developed a UAV and blockchain-based system for inventory and traceability in Industry 4.0 warehouses. While novel, it is reliant on RFID infrastructure, limiting its suitability for environments lacking such setups.

To clarify the positioning landscape, Table 1 provides a comparative overview of common technologies based on accuracy, infrastructure dependency, and scalability. This sets the requirements and trade-offs that motivate the need for new positioning systems.

As shown, most systems that achieve high accuracy (e.g., UWB) require extensive infrastructure, limiting flexibility. Others (e.g., QR codes or stereo vision) are limited by manual steps, line-of-sight constraints, or indoor-only deployment. These limitations motivate the development of alternative systems capable of autonomous relative positioning in unstructured outdoor industrial settings.

Compared to previous works, the main contributions of this article lie in:

- Developing a precise relative positioning framework that is adaptable to dynamic environments.
- Introducing an advanced trustability score to improve spatial reliability and product positioning accuracy.
- Providing a user-friendly digital interface for integration with industrial warehouse management systems.

In summary, this work addresses the need for accurate, infrastructure-light, and scalable product positioning systems suitable for large, metallic, and dynamic storage environments.

## Framework for Self-Product Positioning and Trustworthiness

**Fig. 2:**
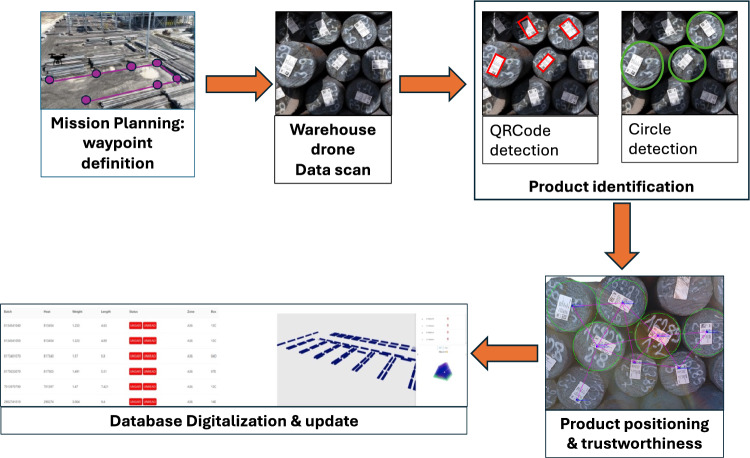
Schematic representation of the developed system in operation within a smart outdoor warehouse

In this work, we propose a framework for building a digital twin of product positions in a warehouse using a camera-equipped drone. This framework also estimates the trustworthiness of each product’s observed position. The system is designed for smart outdoor warehouse environments, where autonomous drones perform flyovers to scan inventory. A schematic of the system is shown in Fig. 2.

The framework consists of five primary stages:

**Mission Planning: Waypoint Definition** The drone’s trajectory is defined as a set of waypoints optimized to cover the scanning area. Waypoints are generated using a greedy sweep-based coverage algorithm and uploaded to the drone via the DJI SDK. The system accounts for flight time limitations and recharges when necessary.

**Warehouse Drone Data Scan** A drone equipped with a 4 K RGB camera (recording at 30fps) captures continuous video frames while navigating its assigned path. Each frame is analyzed to detect both QR codes (used for product identification) and circular markers (used for product position refinement). Algorithm 1 handles the video loop, in which each frame is processed to detect product identifiers (QR codes) and optional markers (circles) used to improve position accuracy. Detections are stored in a dynamic graph structure *G*.

**Algorithm 1:**
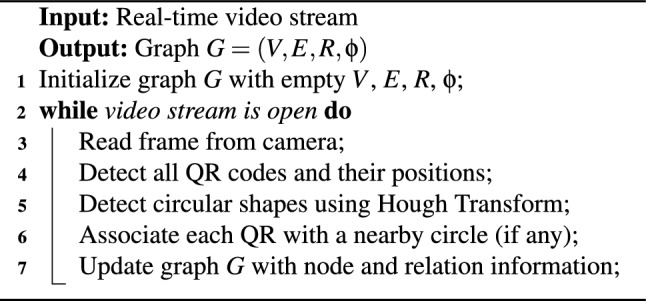
Video Capture and QR Code Detection

Frame preprocessing includes grayscale conversion using OpenCV’s ‘cv2.cvtColor()‘ function and adaptive thresholding via ‘cv2.adaptiveThreshold()‘ to enhance contrast. Noise is reduced using Gaussian blurring. For QR code detection, we use the ZBar-based ‘pyzbar‘ library, chosen for its robustness to partial occlusion and rotation.

Circular markers are detected using the OpenCV implementation of the Hough Circle Transform (‘cv2.HoughCircles()‘), with empirically optimized parameters: ‘dp = 1.2‘, ‘minDist = 20‘, ‘param1 = 50‘, ‘param2 = 30‘, and radius range from 15 to 50 pixels. These parameters were tuned based on test flights to maximize marker accuracy while minimizing false positives.

To improve performance in variable lighting conditions, especially in outdoor settings, histogram equalization was selectively applied to frames with low brightness before QR decoding.

Frame processing latency (from capture to QRCode and marker detection) ranged from 90 to 110 ms, and full graph update cycles remained under 200 ms.

3. **Product Identification** QR codes serve as unique product identifiers. When a circular marker is detected in close proximity to a QR code, the system refines the product’s reported position using the center of the circle (which is more geometrically stable). If no circular marker is found near a QR code, the QR centroid is used directly. Algorithm 2 shows this association and refinement.

**Algorithm 2:**
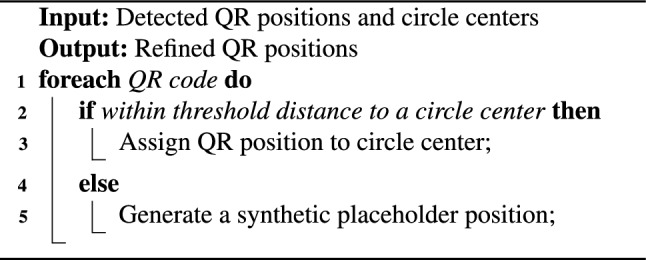
QR Code and Circle Association

4. **Product Positioning and Trustworthiness** After identification, the system computes the relative directions between detected products. For example, if product A is to the left of product B within a single frame, this directional relationship is recorded. The position of each product is defined relative to others using a direction vector, derived from comparing pixel coordinates. A spatial threshold of $\tau = 15$ pixels is used to suppress noise in direction estimation. The core of our system is a directed graph $G = (V, E, R, \phi )$, where (see Algorithm 3):

- *V* is the set of products (nodes),
- *E* is the set of directed edges,
- *R* represents spatial relationships (e.g., RIGHT, LEFT),
- $\phi$ is the trustworthiness score for each observed relation.

**Fig. 3:**
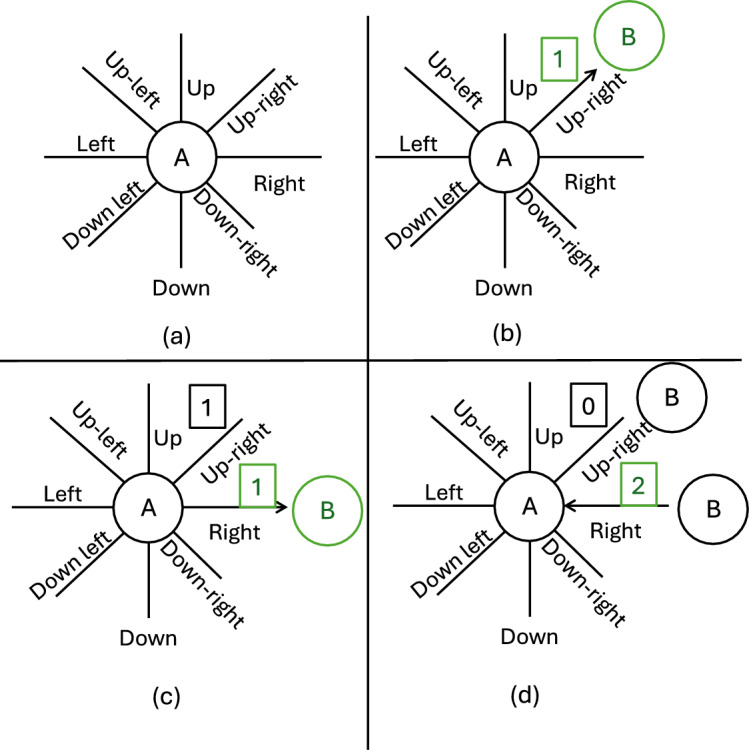
Illustration of an example of trust-ability update [1]

If multiple observations across frames confirm a relation (e.g., A is consistently RIGHT of B), the corresponding edge’s trust score increases. Inconsistencies or missing confirmations reduce the score. Trustworthiness is calculated via a pairwise consistency check: if A says B is RIGHT, but B does not say A is LEFT, the score is penalized (Fig. 3).

**Algorithm 3:**
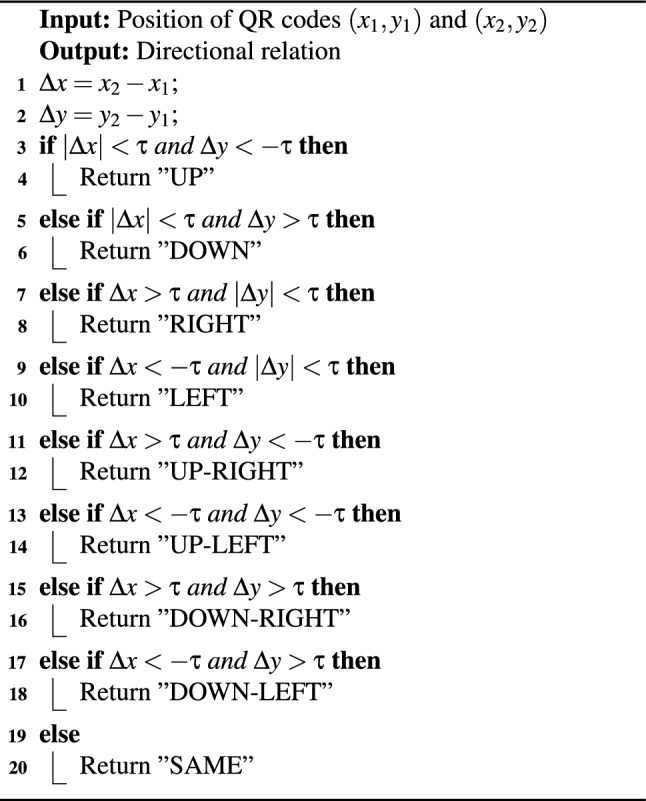
Direction Determination Between Products

5. **Database Digitalization and Update** Once all product positions and relations have been processed, the final product graph *G* is serialized into a JSON format. Each node contains its QR ID, relative positions, and associated trustworthiness scores. The resulting digital twin is uploaded to a central warehouse management system, ensuring the integration with inventory systems.

The entire framework is implemented in Python and optimized for embedded processing. The modular architecture allows future extensions, such as integrating LiDAR sensors or adding SLAM-based geolocation. Most importantly, the inclusion of trustworthiness estimation allows the system to flag unreliable data for human verification, thus increasing operational safety in autonomous warehouse environments.

## Experimental Results

We conducted experiments using a drone equipped with a 4 K camera (30 fps) to capture real-time video data in two types of environments: indoor testbeds and outdoor warehouse-like settings. The drone was programmed to follow predefined waypoints, capturing video sequences from which QR codes were extracted for product localization.

To process the video frames, we applied the preprocess_image() function (see Algorithm 1), which converted RGB images to grayscale and applied adaptive thresholding to enhance QR code visibility. This was followed by the detect_qr_codes() function, which identified QR codes and returned their positions and encoded IDs. Based on these detections, a directed graph representing product relationships was constructed, and a trustability score was calculated.

### Technical Parameters

Key thresholds, such as the acceptable radius for circle detection and the trust aggregation weights, were selected through empirical tuning across multiple scenarios. The detection radius was constrained to a 7–15 cm range to balance sensitivity and specificity. Trust weights favored spatial consistency among neighboring codes. We observed that altering these parameters by ±10–15% had negligible impact on detection accuracy or trustability scores, indicating robustness against minor environmental fluctuations.

The system operates in near real time, with a DJI Dock 2$^{\text {TM}}$ downlink latency of 110–150 ms and a total frame-to-frame delay below 200 ms.

### Indoor Results

**Fig. 4:**
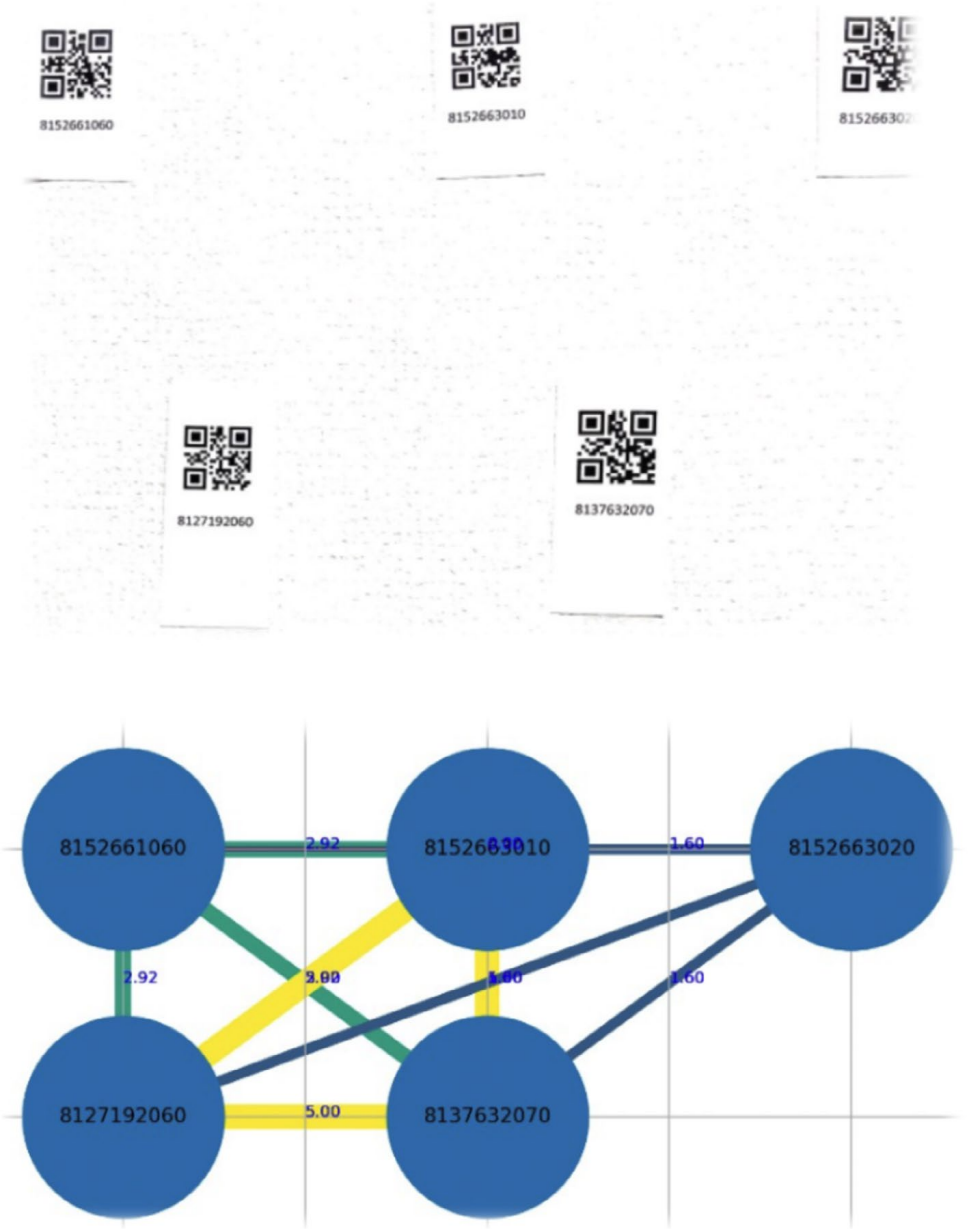
Example of an indoor test: (top) product image, (middle) generated trustability graph, (bottom) user interface with QR-linked data

Figure 4 illustrates a sample indoor test. The top panel shows the product layout, the middle depicts the trustability graph with edge scores, and the bottom shows the user interface with decoded product metadata. Trust scores reflect the confidence of each relative positioning link, computed using the compute_trust() function.

**Table 2:** Indoor test results for varying product quantities

**Products**	**Detected**	**Accuracy (%)**	**Trustability (max 5)**
5	5	100	4.0
22	18	94	4.0
30	25	94	3.8
43	36	94	3.8
90	78	94	3.8

Table 2 presents the performance metrics for each test. *Position Accuracy* refers to the proportion of detected products whose estimated positions are within a 2 cm margin of error from the ground truth. *Trustability* measures the structural reliability of the detected graph, with scores ranging from 0 (unreliable) to 5 (fully consistent). Indoor results show consistent detection performance, with over 81% of products detected and positional accuracy consistently above 94%.

### Outdoor Results

**Fig. 5:**
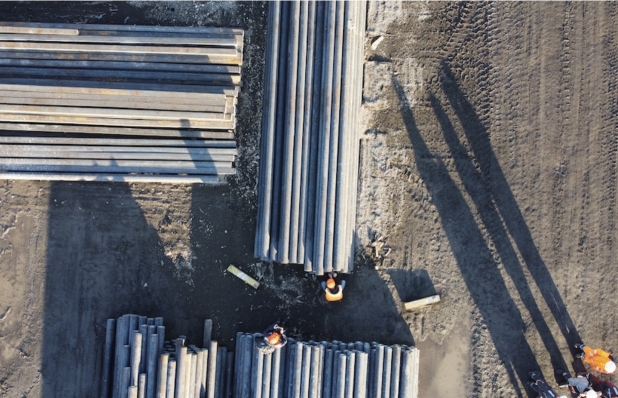
Top view of the outdoor testing site

The outdoor experiments were designed to assess the system’s reliability in more challenging real-world conditions, such as variable lighting and background clutter (Fig. 5).

**Fig. 6:**
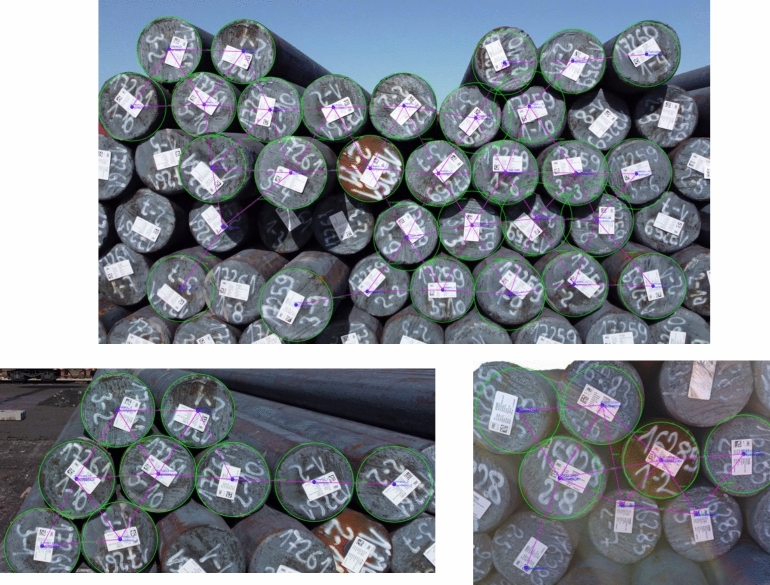
Outdoor QR detection: (left) circle and QR detection; (right) position graph overlay

Figure 6 displays frames from outdoor tests, including visualizations of QR code detections, circle localizations, and the resulting product positioning graph. When both a QR code and a surrounding circle were identified, the system placed the node at the center of the circle. In the absence of a circle, the QR center was used as the positional reference.

**Table 3:** Outdoor test performance across different product volumes

Products	Detected	Accuracy (%)	Trustability (max 5)
4	4	100	4.1
12	12	90	3.9
21	18	83	3.6
67	58	82	3.5
98	87	80	3.3

Table 3 presents average metrics over repeated flights. While the detection and positioning performance remain high, accuracy declines slightly as the number of products increases. This trend is attributed to greater visual complexity, occlusion, and lighting variability.

Despite this, the system demonstrated robustness and scalability without requiring external positioning systems like GPS or UWB. The drone autonomously captured video while the onboard processing maintained consistent QR detection and graph construction.

### Limitations and Detection Robustness

Although the system is resilient to moderate changes in lighting, certain extreme conditions-low light, strong glare, and rain-negatively affect detection. Such environments can cause motion blur, partial occlusion, or poor contrast for QR recognition.

While false positives are rare, false negatives increase when QR codes are damaged or the background is highly reflective. Mitigation strategies include adaptive thresholding with histogram equalization, IR-filtered cameras, or onboard lighting modules. Additionally, deploying multiple drones from varying angles can help mitigate occlusion effects.

### Proposed Improvements and Extended Applications

To improve detection in difficult environments, future work will include training a lightweight Convolutional Neural Network (CNN) for QR localization and restoration. The model will enhance degraded regions before decoding, improving recall under adverse conditions.

In parallel, semantic segmentation (e.g., U-Net with MobileNetV3 backbone) will be introduced to separate QR tags from visually noisy backgrounds, reducing false negatives. Multi-sensor fusion (e.g., stereo depth) will enable context-aware 3D filtering and improve robustness.

Beyond industrial warehouses, the proposed system has potential applications in logistics depots, construction sites, and emergency response zones, offering a fast and infrastructure-light solution for product localization and inventory tracking.

## Conclusion

This article presented a lightweight, vision-based framework for autonomous product detection, positioning, and trust evaluation in industrial warehouses using UAVs. Our system processes real-time drone video to detect QR codes, refines their locations via circle markers, computes directional relations, and aggregates these into a trust-ability graph.

Experimental results showed that the system detected over 81% of products indoors with a positioning accuracy above 94% and average trust-ability scores above 3.0. In outdoor tests, detection rates exceeded 80%, positioning accuracy remained above 80%, and trust-ability scores averaged 3.5, despite challenging lighting and occlusion conditions.

Key parameters such as circle detection radius (7–15 cm) and trust weights were robust to ± 15% variation, and the DJI Dock 2$^{TM}$ downlink latency (110–150 ms) plus 4 K 30 fps camera frame time.

Compared to RFID or GPS methods, our approach requires no fixed infrastructure and adapts readily to dynamic, outdoor environments, making it suitable for applications from construction sites to emergency logistics.

Future work will deal with robustness via lightweight CNNs for QR restoration in low-light and rainy environments, integrate depth sensing for 3D context, and support continuous tracking and ERP integration.

Overall, this framework offers a scalable, infrastructure-free solution for precise, trustworthy product tracking in smart industrial settings, reducing manual effort and enabling data?driven operational efficiencies.

## Data Availability

Not applicable.

## Declarations

### Code availability

All code generated or used during the study is available from the corresponding author upon request.
